# CA-ViT: Contour-Guided and Augmented Vision Transformers to Enhance Glaucoma Classification Using Fundus Images

**DOI:** 10.3390/bioengineering11090887

**Published:** 2024-08-31

**Authors:** Tewodros Gizaw Tohye, Zhiguang Qin, Mugahed A. Al-antari, Chiagoziem C. Ukwuoma, Zenebe Markos Lonseko, Yeong Hyeon Gu

**Affiliations:** 1 School of Information and Software Engineering, University of Electronic Science and Technology of China, Chengdu 610054, China; decmen2008@gmail.com (T.G.T.); qinzg@uestc.edu.cn (Z.Q.); 2 Department of Artificial Intelligence and Data Science, College of AI Convergence, Daeyang AI Center, Sejong University, Seoul 05006, Republic of Korea; 3 College of Nuclear Technology and Automation Engineering, Chengdu University of Technology, Chengdu 610059, China; ukwuoma@std.uestc.edu.cn; 4 Sichuan Engineering Technology Research Center for Industrial Internet Intelligent Monitoring and Application, Chengdu University of Technology, Chengdu 610059, China; 5 School of Life Science and Technology, University of Electronic Science and Technology of China, Chengdu 610054, China; zenebe@uestc.edu.cn

**Keywords:** augmented, contour, CVGAN, enhance, fundus, glaucoma, vision transformer

## Abstract

Glaucoma, a predominant cause of visual impairment on a global scale, poses notable challenges in diagnosis owing to its initially asymptomatic presentation. Early identification is vital to prevent irreversible vision impairment. Cutting-edge deep learning techniques, such as vision transformers (ViTs), have been employed to tackle the challenge of early glaucoma detection. Nevertheless, limited approaches have been suggested to improve glaucoma classification due to issues like inadequate training data, variations in feature distribution, and the overall quality of samples. Furthermore, fundus images display significant similarities and slight discrepancies in lesion sizes, complicating glaucoma classification when utilizing ViTs. To address these obstacles, we introduce the contour-guided and augmented vision transformer (CA-ViT) for enhanced glaucoma classification using fundus images. We employ a Conditional Variational Generative Adversarial Network (CVGAN) to enhance and diversify the training dataset by incorporating conditional sample generation and reconstruction. Subsequently, a contour-guided approach is integrated to offer crucial insights into the disease, particularly concerning the optic disc and optic cup regions. Both the original images and extracted contours are given to the ViT backbone; then, feature alignment is performed with a weighted cross-entropy loss. Finally, in the inference phase, the ViT backbone, trained on the original fundus images and augmented data, is used for multi-class glaucoma categorization. By utilizing the Standardized Multi-Channel Dataset for Glaucoma (SMDG), which encompasses various datasets (e.g., EYEPACS, DRISHTI-GS, RIM-ONE, REFUGE), we conducted thorough testing. The results indicate that the proposed CA-ViT model significantly outperforms current methods, achieving a precision of 93.0%, a recall of 93.08%, an F1 score of 92.9%, and an accuracy of 93.0%. Therefore, the integration of augmentation with the CVGAN and contour guidance can effectively enhance glaucoma classification tasks.

## 1. Introduction

Glaucoma, a leading cause of permanent vision impairment worldwide [1], demonstrates a particularly insidious nature in its initial phases due to the lack of observable symptoms. The number of glaucoma cases escalated to 64.3 million in 2013 and is projected to reach 113 million by 2040 [2]. Regrettably, a significant number of individuals remain oblivious to their condition during the initial phases due to the lack of obvious symptoms. Glaucoma is characterized by intricate changes in the structure of the retina, resulting in considerable thinning of the retina, and the directional reflectivity of the RNFL also decreases with the functional progression of glaucoma [3,4]. This particular trait underscores the critical importance of early detection, as timely intervention plays a vital role in averting visual impairment and irreversible loss of vision [1]. The utilization of fundus photographs serves to delineate the structural attributes of the eye, encompassing the retina, optic disc, macula, fovea, and posterior pole, all of which play a crucial role in visual acuity. The utilization of specialized equipment, such as fundus cameras or scanning laser ophthalmoscopes, is essential for capturing these images, thereby facilitating the identification and monitoring of various fundus disorders [5].

However, the complex nature of initial pathological presentations and the rapid growth in the number of individuals impacted by glaucoma necessitate a significant amount of time and effort for the categorization of this condition, even among proficient ophthalmologists [6]. Hence, the demand for automated methods in glaucoma classification is increasing in order to reduce the number of untreated individuals and alleviate the strain on healthcare professionals, particularly in regions with restricted medical facilities. Notable progress has been achieved in glaucoma classification in the last decade through the utilization of deep learning techniques [5]. Various studies have used convolutional neural networks with fundus images to identify glaucoma [7,8,9]. To address the redundancy issues that affect glaucoma identification, attention mechanisms were later introduced [10,11,12,13]. Additionally, incorporating extra information alongside fundus images has resulted in significant improvements in glaucoma classification [14]. The annotation of the data into three groups was based on the diagnosis made in clinical practice by a glaucoma specialist. The dataset included color fundus photographs and 14 types of metadata (including visual field testing, retinal nerve fiber layer thickness, and cup–disc ratio). Deep learning (DL) was performed first using only the color fundus photographs and then using both the images and metadata, resulting in improved model performance. However, the mentioned methods are suspected to have locality bias and interpretability issues. On the other hand, vision transformers (ViTs) have recently improved the effectiveness of deep learning by incorporating a self-attention mechanism [15,16,17], demonstrating superiority in various medical imaging modalities like computed tomography, X-ray, fundus images [18], ophthalmoscope images [14], and OCT [17]. Several approaches have been employed to improve the classification of glaucoma, but challenges remain due to factors such as limited training data, differences in feature distribution, and sample quality issues. Fundus images display common patterns, recurring features, and slight variations in lesion sizes, which present obstacles in refining glaucoma classification using vision transformers [7]. Prominent entities usually occupy a large percentage of natural photographs and have characteristic features [19]. On the other hand, similar anatomical features and intensity profiles are frequently seen in medical images taken with the same modality, but this is insufficient to differentiate between disorders [20]. Thus, in the context of illness discrimination in medical images, the incorporation of local precise information becomes crucial [21,22]. More focus on key areas and the preservation of fine details in learned representations are necessary to improve the performance of fundus image glaucoma classification [16,23]. Accordingly, numerous endeavors in the field of medical imaging have yielded remarkable successes. However, as far as we are aware, only a few research studies have used the vision transformer (ViT) architecture for the classification of glaucoma utilizing fundus images.

Among them, [3] presented MIL-VT, a modified version of the ViT architecture that leverages features gathered from each patch by integrating a multiple-instance learning head into the vision transformer framework. The method showed slight improvements over CNN methods. Another study [24] employed parameter tweaking in the ViT model and utilized the transfer learning principle. In their tests, the authors pooled five datasets to increase the quantity of training data and included augmentation techniques like flipping and rotation to increase the diversity of the data. They concluded that one crucial point to remember is that ViT models’ superiority over CNNs improves with the diversity and quantity of images [24]. All the presented studies are limited to binary classification and are tested on small sample training datasets. Furthermore, the issues of repetitive characteristics and slight variations in lesion size are not addressed [25]. The employment of transformers indicates a crucial transition toward sophisticated AI methodologies capable of effectively analyzing and deciphering extensive and complex datasets, presenting a promising trajectory for upcoming glaucoma investigations and clinical decision making [23]. Therefore applying vision transformers for glaucoma classification is an emerging and hot topic, and much work is needed in this area. To the best of our knowledge, the utilization of a contour-guided augmented vision transformer for the categorization of glaucoma in fundus images has not been previously investigated. The classification and generalization of multi-class diseases are indispensable not only for accurate diagnosis but also for mitigating training biases. This study introduces a contour-guided augmented vision transformer (CA-ViT) that incorporates contour information and enhances the training dataset through a CVGAN. Contours serve as distinctive features for the foreground of a fundus image, encompassing the optic disc/cup [26]. The integration of contour details significantly boosts model performance by providing accurate boundary delineations that enhance object localization and delineation, resulting in more precise predictions [26]. Geometric and structural details embedded in contours enrich feature representations, thereby reducing false positives and negatives, enabling models to effectively differentiate adjacent objects. This supplementary information enhances model resilience against variations in object shapes and sizes, making classifications more reliable [26]. The CVGAN is used to enhance the training set and dataset for image augmentation and reconstruction. By combining conditional sample generation with reconstruction, this network makes it easier to create features from previously unseen data. Next, we extracted contour information for the training, augmented, and generated images using a contour-guided module. The ViT backbone receives both the extracted contour information and the original images. Accordingly, feature alignment is performed. Finally, during the inference stage, the model categorizes glaucoma into multiple classes. The evaluation of the model’s performance is conducted using only the training, augmented, and generated data, excluding the contour information. The following are the study’s main contributions:

1. We introduce a CVGAN for augmentation to enlarge and diversify the training set of fundus images for glaucoma classification.
2. We propose a contour-guided module to enhance the classification task by extracting the optic disc and optic cup region, where the contour information provides supplementary details to the vision transformer model.
3. We propose a contour-guided and augmented vision transformer framework for multi-class glaucoma classification, enhancing disease diagnosis accuracy. This framework uses a guided contour module to extract detailed optic disc and optic cup features crucial for glaucoma assessment, a ViT backbone to process both the original and contour information, and applies feature alignment to prepare the data for categorization.
4. Rigorous testing and evaluation of the proposed model are conducted using the Standardized Multi-Channel Dataset for Glaucoma (SMDG), which consists of 19 publicly available datasets, such as EYEPACS, DRISHTI-GS, RIM-ONE, REFUGE, combined into a single dataset. This testing demonstrates the superior performance of the proposed model compared to state-of-the-art approaches. Further analysis using t-SNE provides a detailed visualization of the model’s effectiveness in distinguishing between different classes of glaucoma.

This research is structured as follows. Section 2 discusses the related works. Section 3 outlines the materials and methodologies employed. In Section 4, an examination of the experimental configuration and outcomes is provided. Section 5 delivers the results and a discussion. Section 6 concludes this work by summarizing our findings and proposing avenues for future research.

## 2. Related Works

### 2.1. Augmentation and Data Generation

Various methodologies have been explored to address data scarcity challenges in glaucoma diagnosis. Initially, augmentation techniques such as brightness enhancement, contrast modification, rotation, and flipping were utilized; however, these approaches proved inadequate in generating a sufficiently diverse dataset. To alleviate this, various advanced techniques have been introduced but face difficulties due to dataset variability [27]. Consequently, researchers have turned to recent advances in generative adversarial networks (GANs), which have shown promise by offering realistic examples that enhance model training. Unlike conventional geometric modifications, GANs provide learning-based discriminative features, despite their complexity and training challenges [28,29]. Researchers have proposed combining approaches like Conditional GAN (CGAN), Semi-Supervised GAN (SGAN), Auxiliary Classifier GAN (AC-GAN), and Variational Auto-encoder (VAE) with GANs to address data augmentation challenges [28]. These models use class properties to stabilize training and control data generation. Inspired by these techniques, we employ a conditional generative adversarial network model for fundus images, incorporating the auxiliary classifier loss from AC-GAN and ideas from supervised CR-GAN [28,30]. This model enhances the diversity of fundus image datasets by utilizing reconstruction routes and sample creation.

### 2.2. Contour-Guided Approach

This approach uses contour information to guide the model by providing critical supportive information. Contours are utilized as indicative characteristics of image entities, presenting a notable challenge within the field of computational visual perception. The identification of such contours plays a vital role in activities associated with object identification and environmental understanding [26]. This technique has been widely implemented, especially for segmentation purposes in edge-based and region-based approaches [3]. In traditional approaches, the segmentation results depend on the accurate segmentation of edges and regions. However, in deep learning approaches, we have not seen any related works that use the concept of contours for classification problems as additional or supportive information. It is critical to provide contours as supportive information rather than relying solely on the accurate segmentation of contours or regions [26]. Thus far, the application of contour-guided approaches for classifying glaucoma remains unexplored. In our proposed framework, we integrate contour-guided information to address classification challenges. Incorporating contour information can significantly enhance model performance by providing precise boundary details that improve object localization and delineation, leading to more accurate predictions. Contours enrich feature representation with geometric and structural details, reducing false positives and negatives by helping models better distinguish between adjacent objects [3]. This added information increases model robustness against variations in object shapes and sizes, making classification more reliable.

Contouring on fundus images pertains to the extraction of contour-related information from the available images. To facilitate this process, it is imperative to initially preprocess the fundus images. During these phases, it is observed that fundus images frequently display variability in quality, influenced by factors such as lighting conditions, noise interference, and individual anatomical variations. To mitigate these challenges, image normalization methodologies are employed to standardize intensity levels across the entire image. Additionally, noise reduction techniques are implemented through the use of filters, such as Gaussian blur, which aid in smoothing the image while concurrently preserving the integrity of significant structural edges, including those of the optic disc and cup.

The determination of this disc is of critical significance, as it acts as a reference marker for the localization of the optic cup, a minor concavity within the disc that is linked to the advancement of glaucoma. These methodologies operate by identifying areas of rapid intensity variation, which correspond to the edges of the disc. To accurately determine the circular morphology of the optic disc, the Circular Hough Transform method is employed. This methodology is notably effective due to its ability to identify circular forms even amidst noise or partial obstruction. Following the identification of the optic disc, contour extraction is performed to delineate its boundary with a high degree of precision. Thresholding, a segmentation technique based on intensity values, is typically the initial method utilized [26]. This phase serves to segregate the optic disc from the background, thereby facilitating the extraction of its contour. Subsequently, the Sobel operator is employed to discern the contour of the optic disc by enhancing its boundary through the computation of image gradients. The resulting contour delineates the margin of the optic disc, which is essential for subsequent analytical procedures.

The optic cup, located within the confines of the optic disc, represents the next area requiring identification [23]. This commences with the establishment of a region of interest (ROI) within the optic disc. The optic cup is generally characterized by a greater brightness in comparison to the surrounding disc area, making intensity-based segmentation techniques particularly effective [23]. Consequently, we extract an equal number of contour images for all original, augmented, and generated fundus images.

### 2.3. Convolutional Neural Network (CNN)-Based Methods

The detailed survey study presented in [3] explores deep learning techniques for glaucoma detection. In this comprehensive survey study, each of the CNN methods’ strengths and weaknesses has been listed and summarized into six categories. The first category in [7] employs an approach that integrates segmentation and classification using CNNs, which is identified as a strength in improving accuracy and efficiency for glaucoma detection. However, the primary drawback lies in its potential inability to effectively detect glaucoma when used alongside different imaging techniques. The second category [8,31] employs U-Net, CNN, auto-encoder–decoder, and CDR prediction to tackle the identified challenges in glaucoma detection. An advantage of this approach is the fusion of segmentation and CDR prediction using U-Net. Nevertheless, a key drawback is the complexity of the models chosen, leading to difficulties in interpreting their decision-making processes. VGG16, Atrous layers, and Faster RCNN [9,32] aim to address the joint segmentation issue of the OD and OC. A major benefit of this method is the enhanced accuracy achieved through object detection. However, a downside is the potential for decreased accuracy or increased false positives/negatives with varying image quality.

Attention mechanisms with CNNs [10,11,12,13] address the problem of redundancy in fundus images, which affects glaucoma detection. The main strength of this approach is the combination of attention mechanisms with CNNs. While these attention-based CNN models have demonstrated superior performance compared to traditional CNN models, they exhibit shortcomings in effectively capturing significant feature correlations essential for enhancing classification accuracy. Additionally, they result in increased computational burdens. The assessment of pre-trained CNN models and the incorporation of clinical data [1] address the issue of pre-trained CNN model performance and clinical data integration. One key benefit of these methods is the comparative assessment of CNN models. Yet, a drawback of these approaches is the potential bias in linking clinical findings to glaucoma due to variations in diagnostic processes. CNNs, combined with the preprocessing techniques outlined in [18,33], address the challenges of time-consuming glaucoma detection and traditional methods for classifying glaucoma. The primary advantage of these methods is the early and efficient classification of glaucoma with minimal preprocessing. However, a notable limitation is the alteration of certain features or data crucial for disease detection.

CNNs with different variants of ResNet, as described in [5,34], effectively address the challenges of reducing false positives and enhancing overall detection accuracy. One notable advantage of these methods is their ability to precisely classify glaucoma based on fundus images. Nevertheless, a drawback of such approaches lies in their high training complexity, compounded by the potential variability of fundus images due to factors like racial background, age, or the imaging tools utilized. Another method presented to solve the problem of efficient and effective glaucoma detection is CNN-based InceptionV3 [35]. The strength of this type of approach is its resulting higher accuracy and AUC compared to other algorithms.

In a recent study [25], the researchers used fundus images to propose an improved self-attention-directed network and adapter for multi-stage glaucoma classification. The authors proposed using an upgraded CNN framework, AES-Net, and an inventive adaptor to accurately categorize glaucoma phases. In particular, they suggested using a spatial adapter module to create more robust feature representations and an enhanced self-attention module (ESAM) to record global feature correlations between the pertinent channels and spatial coordinates. However, in order to test their suggested model, they only employed small and unbalanced datasets. Moreover, the suggested AES-Net requires a fairly large number of learnable parameters, which increases computational costs.

All of the aforementioned efforts are based on CNN techniques. However, they are limited to binary classification or a maximum of three classes due to their use of small and restricted dataset samples. This restriction makes it difficult to determine how severe the condition is at different points in time. The augmentation methods used, including flipping and horizontal and vertical transformations, do not significantly improve the dataset’s variety and therefore do not provide the model with enough diverse examples to learn from during training. Furthermore, there is no strategy that can handle the similar characteristics of fundus images across multiple stages. Lastly, the majority of CNN techniques are not as interpretable as ViT models.

### 2.4. Vision Transformer (ViT)-Based Methods

Several studies have been conducted using transformer concepts applied to medical image classification problems [15,27,36,37,38]. However, for glaucoma classification problems using fundus images, only a few studies have been conducted so far. Among them, the reviewed research conducted in [39,40] provided a thorough comparison of CNN and ViT models using fundus image modalities. The study included extensive experiments and analyses involving pure CNN-type models, hybrid models, and ViT models. The researchers highlighted that the selection of models should be based on the specific problem and objectives one intends to accomplish. The most recent study by [17] investigated a spatial-aware transformer GRU framework developed with the aim of enhancing the diagnosis of glaucoma through the utilization of 3D OCT imaging. The researchers employed the ViT large model for feature extraction and incorporated a bidirectional gated recurrent unit to efficiently capture inter-slice spatial relationships The study conducted by [41] on the utilization of the ViT architecture for object detection within the domain of computer vision revealed significant challenges. The investigation examined the possibility of enhancing the vision transformer for object detection in medical imaging, focusing specifically on the identification of glaucoma. The researchers employed the SMDG dataset to evaluate object detection related to the optic disc and optic cup.

In the study conducted by [3], the MIL-VT was introduced as a modified iteration of the ViT architecture, effectively incorporating characteristics obtained from individual patches through the inclusion of a multiple-instance learning head within the vision transformer framework. Furthermore, the researchers pre-trained the model utilizing various fundus imaging datasets to initialize it, and then they fine-tuned it specifically for the categorization of retinal diseases. The suggested method added no new layers or modified any hyperparameters; it stayed true to the original ViT design. Test findings on multiple datasets showed a marginal improvement in performance over some of the most advanced convolutional neural networks (CNNs). Another study [24] employed parameter tweaking in the ViT model and utilized the transfer learning principle. In their tests, they pooled five datasets to increase the quantity of the training data and included augmentation techniques like flipping and rotation to enhance the variety within the dataset. They concluded that one crucial point to remember is that ViT models’ superiority over CNNs improves with the diversity and quantity of images [24].

Current state-of-the-art works have been limited to the binary classification of glaucoma, which does not clearly show the severity level of the disease. The augmentation mechanisms lack the ability to produce diverse fundus images. Additionally, there is a lack of identifying repetitive characteristics of fundus images, which influences the learning of discriminative features for glaucoma. To mitigate these issues, we propose a contour-guided and augmented vision transformer. This approach is motivated by the ability of contour methods to accurately extract the required edges and regions, as demonstrated in [19]. With the availability of the SMDG dataset [42], we are also inspired by [29] for synthetic data generation of fundus images and by [43] for a simple, efficient, and interpretable transformer model for fine-grained images.

## 3. Materials and Methods

### 3.1. Materials

The Standardized Multi-Channel Dataset for Glaucoma (SMDG-19) compiles and standardizes 19 public glaucoma datasets. It includes full fundus images, image metadata (such as blood vessel and optic disc segmentation), and per-instance text metadata like age and sex. This dataset is the largest available collection of fundus images featuring glaucoma [42]. We utilized all the available fundus images from the different datasets, totaling 11,665 images. These include BEH with a total of 79 fundus images, CRFO with a total of 634 images, JSIEC with a total of 49 images, DRISHTI-GS with 101 images, EyePacs with 3270 images, ODIA-ODIR with 4662 images, REFUGE with 759 images, and others, as shown in Table 1. Fundus images are categorized into five classes: normal, suspect, PAOG/NTG, referable, and glaucoma.

As mentioned earlier, a total of 11,665 fundus images from [42] were obtained before applying augmentation and data generation using CVGAN.

### 3.2. Methods

#### 3.2.1. Data Preprocessing

In the data preprocessing stage, we first processed the fundus images to ensure they were ready for analysis. We split the dataset into training and testing sets, with 70% of the dataset used for training, while the remaining 30% was set aside for validation and testing. Then, we resized the fundus images to a 512 × 512 resolution. Diverse data augmentation strategies were employed to combat issues related to overfitting and data imbalance. These techniques included transposition, flipping, rotation, random adjustment of brightness, blurring, distortion, contrast enhancement, and the application of limited adaptive histogram equalization. In addition, we extracted an equal amount of contour information from each given fundus image. Lastly, besides using ordinary augmentation techniques, CVGAN-based augmentation was used to enlarge the training dataset and generate plausible fundus images. After the augmentation and data generation procedures, the collective training dataset encompassed 24,127 images. The preprocessed data were utilized across all experimental endeavors involving the model.

#### 3.2.2. Overview of the Proposed Method

**Figure 1:**
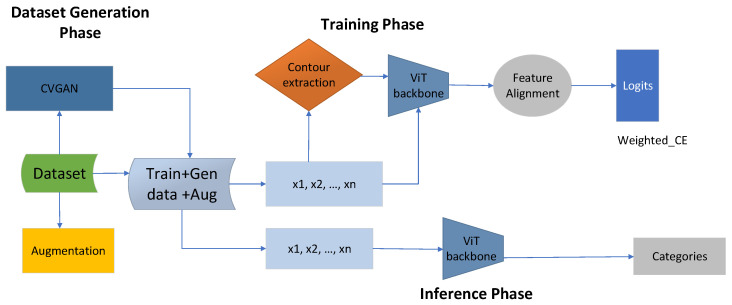
The proposed contour-guided and augmented vision transformer framework has three main phases. The first phase is the data generation phase, during which the dataset is enriched using CVGAN and augmentation, producing a large dataset with training, augmented, and generated data. During the training phase, the total dataset Xt={Aug+Gen} is the sum of the augmented and the generated data. We then extract an equal amount of contour information from the total available dataset, denoted as xt. We extract Xc={x1,x2,…,xn}, and this information is given to the ViT backbone. The ViT backbone processes both the contour information and original images as $X_{total}=\left\{Aug+Gen\right\}$, which is then given to feature alignment, where the weighted cross-entropy loss is calculated as $L_{WCE}=-\sum_{i=1}^{N}w_{i}y_{i}\log\left({\hat{y}}_{i}\right)$. The last stage is the inference phase, where the original images with $X_{total}=\left\{Aug+Gen\right\}$ are given to the ViT backbone to perform categorization or classification.

The established framework was developed to facilitate end-to-end classification of glaucoma through three phases: the data generation phase, the training phase, and the inference phase. In the first phase, we generate data using both augmentation techniques and CVGAN approaches (Figure 1). The final dataset is the collection of $X_{total}=\left\{Train+Aug+Gen\right\}$. In the second phase, we train the proposed model by providing both the original data as $X_{total}=\left\{Train+Aug+Gen\right\}$ and the supplementary guidance information, which is the contour data. By extracting contour information from the total dataset $X_{total}$, we obtain an equal amount of contour information, denoted as Xc={x1,x2,…,xn}, and both are provided to the ViT backbone. In the inference stage, the categorization of glaucoma is performed using the ViT backbone.

#### 3.2.3. Problem Formulation

Our goal is to enhance glaucoma classification by using the current state-of-the-art deep learning model—the vision transformer. This is mainly due to factors such as scarcity of training samples, feature distribution, and overall sample quality. Furthermore, fundus images demonstrate significant similarity, repetitive characteristics, and slight variations in lesion size, making it challenging for the vision transformer to enhance glaucoma classification. Therefore, we enrich the dataset size and diversify its distribution using the CVGAN. Assume we have an original dataset represented as $X=\{x_{1},x_{3},\ldots ,x_{n}\}$; we obtain an augmented version of this set as $Aug=\{aug_{1},aug_{3},\ldots ,aug_{n}\}$, and a generated version as $Gen=\{gen_{1},gen_{3},\ldots ,gen_{n}\}$, so the total dataset is $X_{total}=\left\{Aug+Gen\right\}$, which is the sum of the augmented and generated datasets. Then, we obtain an equal amount of contour information from the total available dataset, denoted as Xt. We extract $Xc=\{Xt_{1},Xt_{2},\ldots ,Xt_{n}\}$. Most previous studies are based on binary classification problems, which indicate either the presence or absence of glaucoma. Let us assume the disease *d* has several severity levels $S=\{S_{1},S_{2},\ldots ,S_{n}\}$; this means previous approaches are limited to two classes only, where $S_{1}$ represents the presence of glaucoma and $S_{2}$ represents normal. Consequently, they missing the severity details of disease *d*, such as suspect, referable, and POAG/NTG classes. In addition, fundus images are complex, making it difficult to identify optic disc and optic cup regions due to their similar characteristics without providing additional information. Let $X_{i}$ represent the original fundus images, with region information $(x_{\mathrm{region}}$) and edge information $(x_{\mathrm{edge}}$). Without providing additional information, identifying critical areas of the disease is difficult. Therefore, we provide both Xt and Xc to the ViT backbone. Feature alignment on the ViT backbone is performed using $L_{WCE}$, attained by the summation $L_{WCE}=-\sum_{i=1}^{N}w_{i}y_{i}\log\left({\hat{y}}_{i}\right)$, where $w_{i}$ refers to the class weights based on the distribution of the dataset. In the inference stage, we only use the original images $X_{total}=\left\{Aug+Gen\right\}$. Here, we do not use the contour information during the evaluation of the model’s performance. In the inference stage, the model is evaluated using the training, augmented, and generated original images, and then categorizes them into five classes: Class={glaucoma,normal,POAG/NTG,referable,andsuspect}.

#### 3.2.4. Data Generation and Augmentation

**Figure 2:**
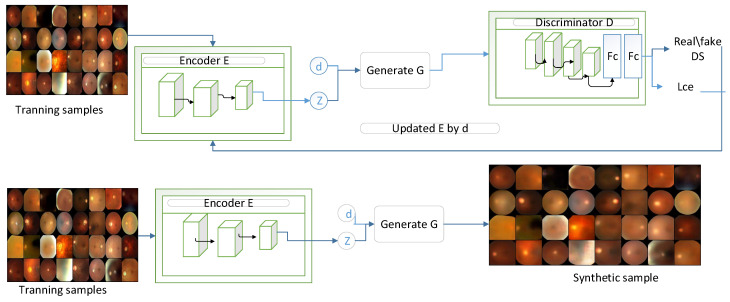
CVGAN sample reconstruction network.

In the preliminary stage, our primary aim is to tackle the issue of limited data availability by creating fundus images to enhance the diversity and scale of the dataset, making it appropriate for the transformer model. This goal is accomplished by utilizing a CVGAN in the generation process (Figure 2), which consists of two distinct pathways, each with unique capabilities. To enhance the training dataset for glaucoma fundus images, a CVGAN image reconstruction network was developed, drawing inspiration from [28]. The encoder *E* generates a latent feature $\bar{z}$, the generator *G* creates a synthetic sample $\bar{x}$, and the discriminator *D* differentiates $\bar{x}$ from the real sample *x*. The encoder E(x) processes the input training sample *x* to generate an encoded latent feature space $\bar{z}$ that preserves identity and an estimated class label $\bar{d}$ for glaucoma.

(1) $$\left(\bar{d},\bar{z}\right)=\left(E_{d}\left(x\right),E_{z}\left(x\right)\right)=E\left(x\right)$$

The feature space $\bar{zi}$ and label disease di are consequently employed as inputs for the generator $G\bar{zi}$, di to generate a synthetic sample $\bar{si}$, which acts as a representation of *x*. The optimization of *G* is not directly conducted by De but rather indirectly through the ED network. By incorporating the real/fake $D_{s}$ loss, the cross-entropy $L_{CEL}$ loss from the De network, and the loss of the predicted labeled class $\bar{di}$ from ED itself, the ED network strives to improve the generation of realistic synthetic samples by optimizing *G*. Subsequently, $D_{s}$ differentiates between the original sample *x* and the synthetic sample $\bar{x}$. Furthermore, the De network incorporates a gradient penalty $L_{GP}$ to ensure stable training of GANs, in accordance with the methodology presented in [28].

Based on [44], $\hat{xi}$ is the estimation derived from the interpolation of authentic data samples xi and generated samples $\bar{xi}$, expressed by the following mathematical expression:

(2) $$\hat{xi}=\epsilon \times xi+\left(1-\epsilon \right)\times \bar{xi}$$

A value is selected at random and falls within the range of 0 to 1. The D network anticipates the performance of the G generation by utilizing an auxiliary classifier cross-entropy loss (LCE) for the class label d, which aims to optimize the E network. This process evaluates the performance of class labels in generated images.

(3) $${\bar{L}}_{CEL}=\frac{\exp(mf\left(x,\theta_{f}\right))}{\sum_{x}\exp\left(mf\left(x_{k},\theta_{f}\right)\right)}$$

(4) $${\bar{L}}_{CEL}={\bar{L}}_{CEL1},\ldots ,{\bar{L}}_{CELn}$$

(5) $$L_{CEL}=-{\bar{L}}_{CEL}\log\left({\bar{L}}_{CEL}\right)$$

The auxiliary classifier cross-entropy loss $L_{CEL}$ pertains to *W*, denoting weight, in the context where $fx,\theta_{f}$ represents a functional relationship involving the actual instance *x* and the parameter θ for learning. During the training phase, the neural network *G* utilizes the class label di of the disease to guide it towards a common feature through the reduction of the discriminator loss $L_{D_{c}}$.

(6) $$L_{D_{c}}=E_{x_{i}∼p_{x}}\left[D_{s}\left(\bar{x}\right)-D_{s}\left(x\right)\right]+L_{GP}-\lambda_{2}L_{CEL}$$

From the Ge network, the discriminator real/fake loss $D_{s}$, the synthetic sample $\bar{xs}$, the gradient penalty loss $L_{GPL}$, and the De network cross-entropy loss $L_{CEL}$ are represented by Equation (7). The optimized latent feature $\bar{z}$, learned from the discriminator’s $D_{s}$ and $L_{CEL}$ losses, is provided by the *E* network to optimize the Ge network through maximizing the $E_{d}$ loss $L_{E}$.

(7) $$L_{E_{d}}=E_{x∼p_{x}}\left[D_{s}\left(\bar{x}\right)+\lambda_{4}p\left(D_{d}\left(\bar{x}\right)=d\right)-\lambda_{5}L_{1}\left(x,\bar{x}\right)-L_{CEL}\right]$$

The $L_{1}$ loss ensures that $\bar{x}$ serves as the reconstruction of *x*. The constants $(\lambda_{1}=10$), $\lambda_{2}=\lambda_{3}=\lambda_{4}=\lambda_{5}=1$ are utilized to regulate the relative loss weight in the objective function, as explained in WGAN-GP [44]. The goal of the target function in the comprehensive sample reconstruction network is centered around the amalgamation of losses *E* and *D*.

(8) $$L_{CVGANbased}=L_{E_{d}}+L_{D_{c}}$$

After training the reconstruction network in the training phase, the training set is enlarged by newly generated synthetic samples generated during the generation phase.

As indicated in Table 2, a total of 24,127 fundus images, including CVGAN-generated and augmented images, were used. A total of 4035 fundus images—specifically, glaucoma=1000, normal=2000, referable=1000, suspect=123, and POAG/NTG=140—were generated using CVGAN-based augmentation, as shown in Table 2. The generator methodologies facilitate the creation of synthetic images based on the input data [29]. In this case, the input data pertain to the SMDG dataset, which consists of three dominant classes, resulting in the generation of images predominantly showcasing these major classes. Synthetic data generation introduces a broader spectrum of variations than standard augmentation, reducing the risk of overfitting on limited datasets [22]. This ensures that the ViT model encounters a diverse set of scenarios during training, enhancing its ability to generalize to new unseen data.

#### 3.2.5. Contour-Guided Module

In the dataset, which encompasses training, augmented, and generated fundus images, we extract an equal amount of contour information from the total available dataset, denoted as Xt. We extract $Xc=\{Xt_{1},Xt_{2},\ldots ,Xt_{n}\}$. We follow specific steps and algorithms in order to extract contour information. To achieve this objective, the first step entails applying a smoothing process to the input image, followed by employing a Sobel filter to aid in edge detection within the image. Subsequently, a non-maximum suppression technique is implemented to selectively retain local maximum pixels oriented in the gradient direction while simultaneously suppressing the remaining pixels. Thresholding is then applied to eliminate pixels falling below a specified threshold while preserving those surpassing a certain threshold to eliminate spurious edges resulting from noise. Finally, hysteresis tracking is conducted to enhance the strength of a pixel if any of its eight neighboring pixels exhibit high strength [26]. Given an image $x\in R^{H\times W\times C}$ with spatial resolution *H*, *W*, and number of channels *C*, the image is first converted to grayscale if it is not already. The grayscale image $x_{gray}\in R^{H\times W}$ is then subjected to edge detection algorithms, such as the Canny edge detector, to highlight the edges. After that, noise reduction is applied. Gaussian blur is used to smooth an image and remove noise. This is accomplished by applying the image convolution technique with a Gaussian kernel, represented as Kernel={3∗3,5∗5,7∗7…,etc}. Next, we use the Sobel kernel in both the horizontal and vertical directions on the smoothed picture to obtain the first derivative in the vertical direction Gy and the horizontal direction Gx. The edge gradients *G* and θ are then computed as shown below.

(9) $$Edge_{gradient}\left(G\right)=\sqrt{G_{x}^{2}+G_{y}^{2}}$$

(10) $$\theta =\tan^{-1}\left(\frac{G_{y}}{G_{x}}\right)$$

**Figure 3:**
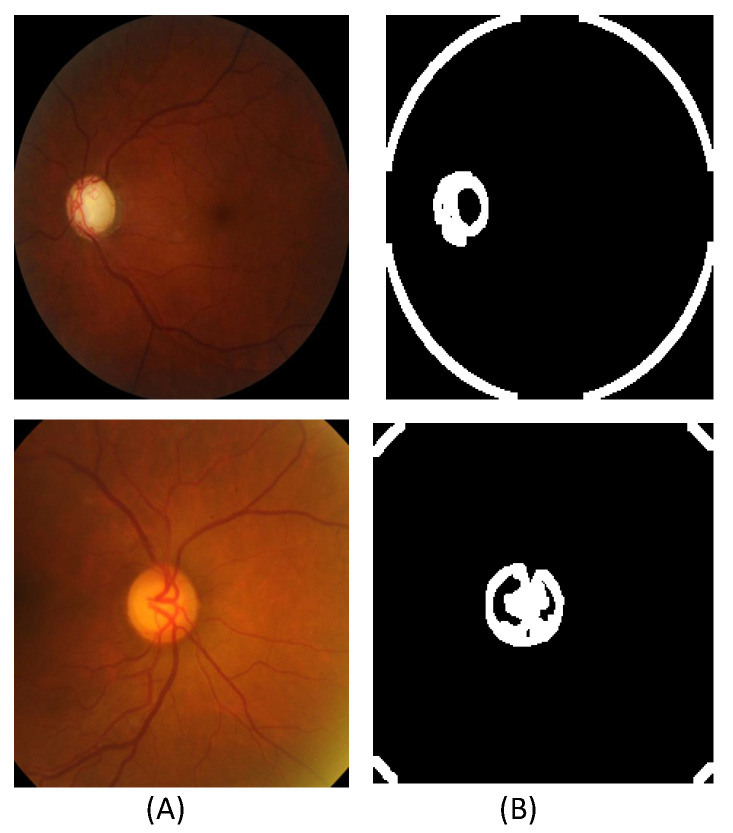
Extracted contour sample images from the SMDG dataset: (**A**) The original fundus images. (**B**) The extracted contour images.

We proceed with the process of eliminating any superfluous pixels that do not make a significant contribution to the formation of edges. To accomplish this objective, each pixel is thoroughly examined in relation to the gradient to determine whether it meets the criteria to be considered a peak within its immediate vicinity. Upon the detection of a pixel as a local maximum, it is subsequently classified as a potential candidate for further examination; conversely, if it fails to satisfy this criterion, its value is reset to zero (Figure 3).

#### 3.2.6. Feature Alignment and the Loss Function

Feature alignment is performed during the training phase. During this phase, the ViT backbone processes both the input of the original image and the contour information for training data. The total dataset $X_{total}=\left\{Aug+Gen\right\}$ is the sum of the augmented and generated datasets. The total number of contours is obtained as $X_{total}=\left\{Xt_{1},Xt_{2},\ldots ,Xt_{n}\right\}$. Inspired by feature fusion [45] to obtain more robust features for glaucoma classification, we combine the original image features with the contour features extracted by the ViT backbone as follows:

(11) $${\hat{y}}_{i}=ViT\left(X_{c}\right)+ViT\left(X_{i}\right)$$

Given the aligned features ${\hat{y}}_{i}$, to adjust the loss based on the frequency of each class, we introduce a weight $w_{j}$ for each class *j*. Overall, we calculate the loss function with class-imbalance awareness for glaucoma classification, $L_{\mathrm{WCE}}$, as

(12) $$L_{\mathrm{WCE}}=-\frac{1}{N}\sum_{i=1}^{N}\sum_{j=0}^{C-1}w_{j}\cdot 1\left(y_{i}=j\right)\log\left({\hat{y}}_{ij}\right)$$

where $y_{ij}$ is the predicted probability for the *i*th sample belonging to class *j*, $y_{i}$ is the true class label for the *i*th sample, and $1(y_{i}=j)$ is an indicator function that is 1 if $y_{i}=j$ (i.e., the true class is *j*) and 0 otherwise.

#### 3.2.7. Inference Phase

The last phase of the proposed model is the inference phase. In this stage, only the original images, consisting of $X_{total}=\left\{Aug+Gen\right\}$, are given to the ViT backbone to determine the categorization. Therefore, given the discriminative spatiotemporal features ${\hat{z}}_{i}^{c}$ from the ViT backbone $f_{q}$, we further transform them using the inference phase $y_{i}=F_{q}\left({\hat{z}}_{i}^{c}\right)$. We then calculate the weighted cross-entropy loss [46] $L_{WCE}$ with *N*-class cross-entropy loss using the following equation:

(13) $$L_{WCE}=-\sum_{i=1}^{N}w_{i}y_{i}\log\left({\hat{y}}_{i}\right)$$

The detailed model architecture used in CA-ViT, described below, includes the architecture of both the CVGAN and CA-ViT models, as presented in Table 3 and Table 4, respectively. These tables provide a clear breakdown of the models’ architectures, including the number of layers, types of layers, and hyperparameters used.

## 4. Experimental Setup

For the experimental setup, to ensure a fair comparison, we used the same environment across all models. We used the same dataset, augmentation strategy, batch size, and number of epochs for both the state-of-the-art (SOTA) models and the proposed model. In addition, to make the comparison more valid, we conducted ablation studies by adding and removing modules in the proposed vision transformer model. Further training parameters can be found in Table 5. The dimensions of the input were modified to 512 × 512 pixels. The studies were carried out on a machine with four NVIDIA GeForce RTX 3080Ti GPUs, each with six gigabytes of RAM, using Python 3.8.16 and the PyTorch 1.13.1 DL library. A CUDA version 11.7 NVIDIA GeForce RTX 3060 laptop GPU and a 12th-generation Intel (R) CoreTM (TM)-i9-12900H CPU were also included in the system.

Various assessment criteria were utilized to validate the effectiveness of the proposed method in classification tasks, encompassing metrics such as accuracy (Acc), precision (Pre), recall (Rec), sensitivity (Sen), specificity (Spe), and F1 score (F1). The F1 metric assesses accuracy by computing the harmonic mean of Pre and Rec [47]. Accuracy, denoted as Acc, represents the proportion of individuals with glaucoma compared to those without the disease.

**Accuracy (Acc)**

(14) $$Acc=\frac{TP+TN}{TP+TN+FP+FN}$$

**Precision (Pre):**

(15) $$Pre=\frac{TP}{TP+FP}$$

**Recall (Rec) or Sensitivity (Sen):**

(16) $$Rec=Sen=\frac{TP}{TP+FN}$$

**Specificity (Spe):**

(17) $$Spe=\frac{TN}{TN+FP}$$

**F1 score (F1):**

(18) $$F1=2\times \frac{Pre\times Rec}{Pre+Rec}$$

## 5. Results and Discussion

In this section, the results from the experiments are presented along with a discussion. Two approaches are used to examine and describe the findings. The first approach is quantitative and makes use of a number of assessment metrics, including accuracy, precision, recall, and F1 score. Furthermore, training and validation accuracy graphs, as well as confusion matrices, are displayed. The second approach is qualitative and makes use of t-SNE analysis and attention heat maps. Furthermore, ablation studies are introduced and reviewed.

The model complexity comparisons between various CNN and ViT models, as shown in Table 6, indicate that CNN models are relatively lightweight and require fewer parameters compared to ViT models. ResNet50 is efficient in terms of FLOPs and parameters but achieves lower accuracy. In contrast, ViT16, DeiT, and Swin have higher FLOPs and parameters due to their transformer architecture, leading to moderate accuracy. CaiT strikes a balance between parameters and FLOPs with reasonable accuracy. Finally, our model achieves the highest accuracy with relatively fewer parameters and FLOPs, demonstrating high computational efficiency. Therefore, the simplicity and interpretability of the proposed model enable its utilization without extensive computational resources, in contrast to other ViT models. In terms of performance, superior outcomes were achieved with the utilization of the suggested model, as depicted in Table 6. Thus, its preference is justified by the combination of high performance results and its lightweight design.

**Figure 4:**
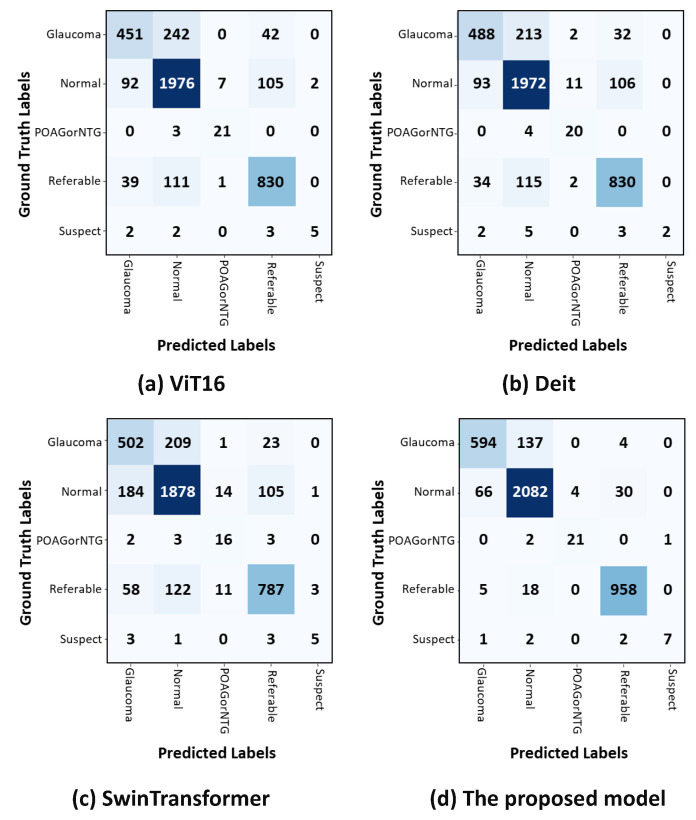
Confusion matrices for classifying glaucoma using test datasets. The actual and anticipated outcomes for each of the five classes are displayed in the confusion matrices. (**a**) The ViT model’s confusion matrix. (**b**) DeiT confusion matrix. (**c**) Swin transformer confusion matrix. (**d**) CA-ViT confusion matrix.

The confusion matrix in Figure 4a shows that in the POAG/NTG classification, the ViT achieved the highest results with 87.5%, whereas in the glaucoma classification, it attained the lowest results with 61%. Figure 4b shows that DeiT achieved the highest results in the POAG/NTG classification with 83%, whereas it faced challenges in classifying suspect classes, attaining the lowest results with 46%. The confusion matrix in Figure 4c demonstrates the proposed method’s competitive classification performance. In the POAG/NTG class, the proposed approach achieved the lowest classification results (68%), but it more accurately identified referable cases (80%). In Figure 4d, it can be seen that the proposed model (CA-ViT) surpassed all other approaches in classifying all classes of glaucoma. The attained results for classifications of normal (95%) and referable (97%) were high, and it demonstrated the lowest performance (80%) in the identification of the glaucoma class. The confusion matrix presented in Figure 4 indicates that the number of fundus images within each class had a great impact on the accuracy achieved. The number of fundus images was very small for classes like suspect and POAG/NTG, resulting in lower confusion matrix values compared to the other three classes—specifically glaucoma, normal, and referable—which had higher numbers of fundus images. Although this outcome reflects the reality and robustness of the proposed model, balancing the class distribution is a good option to consider.

Table 7 shows a statistical comparison of the evaluation metrics, including precision, recall, F1 score, and accuracy. The proposed model achieved a precision of 93.0%, significantly higher than the 72.2% achieved by ResNet50 and the 73.4% achieved by ViT16. Similarly, the proposed model’s recall was 93.0%, compared to 72.1% for ResNet50 and 73.4% for ViT16. The F1 score for our model was 92.9%, much higher than the 72.2% achieved by ResNet50 and the 73.2% achieved by ViT16. Finally, in terms of accuracy, the proposed model achieved 93.0%, surpassing the 72.81% achieved by ResNet50 and the 73.31% achieved by ViT16. These values demonstrate that the proposed model outperformed traditional CNN and ViT methods across all evaluation metrics, indicating its superior performance in accurately classifying fundus images. This improvement is due to the innovative integration of contour and data generation methods.

**Figure 5:**
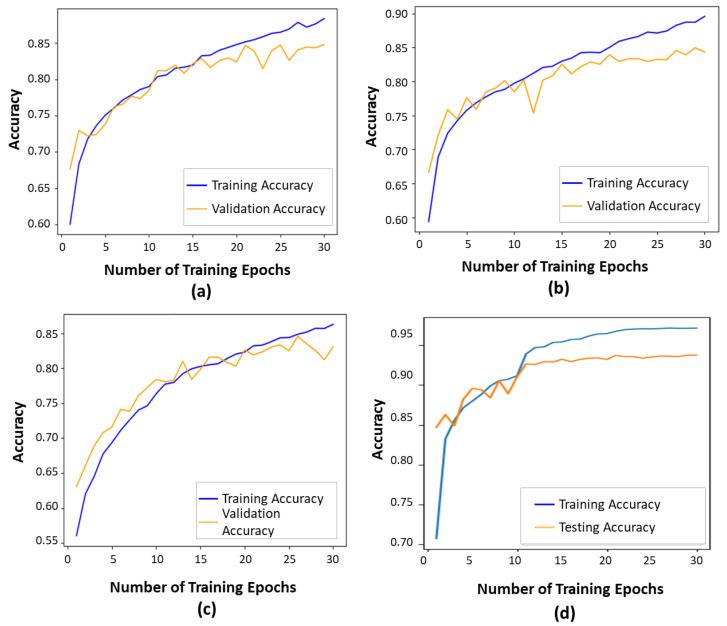
Training and validation accuracies show how well a deep learning model performs during training and generalizes to unseen data, respectively: (**a**) The ViT16 model exhibited a training accuracy of 87% and a validation accuracy of 81%. (**b**) The DeiT model exhibited a training accuracy of 89% and a validation accuracy of 81%. (**c**) The Swin transformer exhibited a training accuracy of 86% and a validation accuracy of 78%. (**d**) The proposed model.

As presented in Figure 5, the contour-guided and augmented vision transformer outperformed the state-of-the-art models in both training and validation accuracies. This indicates that the proposed model learned effective patterns as training progressed, achieving a training accuracy of 97% and a validation accuracy of 93.6%. Compared to the ViT16 model, the proposed model showed an improvement of 14% in validation accuracy and 4% in training accuracy. When compared to the DeiT transformer, we observed improvements of 12% in validation accuracy and 4% in training accuracy. Finally, compared to the Swin transformer, we observed improvements of 15% in validation accuracy and 7% in training accuracy. Overall, this shows that the proposed model learns better features than the SOTA models, making it a more effective strategy for improving glaucoma classification through optimal performance outcomes.

### 5.1. Ablation Studies

To comprehend the impact of each component of the proposed framework, we conducted extensive ablation experiments on the SMDG dataset [42]. To the best of our knowledge, and since there was no baseline model, we used [43] ViT+A as the baseline model and excluded the added components. Then, we assessed the impact of each of the following separately: the contour-guided module and the CVGAN-based augmentation technique.

#### 5.1.1. Effect of CVGAN-Based Augmentation

Table 8 shows the effect of using CVGAN for augmentation purposes. To do this, we first assessed the baseline ViT model without incorporating the generated images from CVGAN. While isolated augmentation techniques provide essential data diversity for training robust deep learning models, incorporating a synthetic data generator offers significantly greater diversity. This is crucial for the vision transformer (ViT) model, as it results in superior performance [28]. As presented in Table 8, the proposed method enhanced performance by 3.7%, 4.1%, 4.0%, and 3.8% in terms of precision, recall, F1 score, and accuracy, respectively. Similarly, the *p*-value of <0.05 indicates a statistically significant improvement when adding the data generator to the ViT + G model. This indicates that using CVGAN for augmentation purposes increases the sample size for training. The diverse dataset had a significant impact on enhancing glaucoma classification by improving the performance of the ViT + A model.

#### 5.1.2. Effect of Contour Mechanism

To evaluate the efficacy of the contour mechanism in acquiring discriminative representations of the optic disc and the optic cup for the classification of glaucoma, we trained the proposed model both with and without the contour module. As shown in Table 8, the contour-guided vision transformer (ViT) model reveals a statistically significant difference, with the results from the ViT model lacking the contour module (ViT + A) being substantially inferior to those from the ViT model that integrates the contour mechanism (ViT + C). Moreover, as shown in Table 8, the proposed methodology resulted in improvements in performance metrics of 2.4%, 1.7%, 2.6%, and 2.0% with respect to precision, recall, F1 score, and accuracy, respectively. We computed a *p*-value of less than 0.05, which indicates a statistically significant enhancement attributable to the incorporation of contour information into the ViT + A model. This observation suggests that the integration of contour information had a substantial influence on improving glaucoma classification, as evidenced by the superior performance outcomes of the ViT + C model compared to those of the ViT + A model.

#### 5.1.3. Combined Effects of Augmentation, Generation, and Contour

The ViT [43] model is based on a pre-trained ViT model using DETR [48]. We assessed its impact by including and excluding the fine-tuning mechanism and found a significant difference in the performance of the model. Finally, we assessed the combined impact of all attributes—augmentation, generation, and contour—in the proposed CA-ViT model. When we evaluated the combined impact, the model’s performance was boosted by 8% across all performance metrics. It is highly likely that training on a large pre-trained image set improved glaucoma classification accordingly. The CA-ViT model, described as ViT + A + G + C in Table 8, had a *p*-value of <0.01, indicating a highly significant improvement when combining all attributes—augmentation, generation, and contour.

### 5.2. Qualitative Analysis

**Figure 6:**
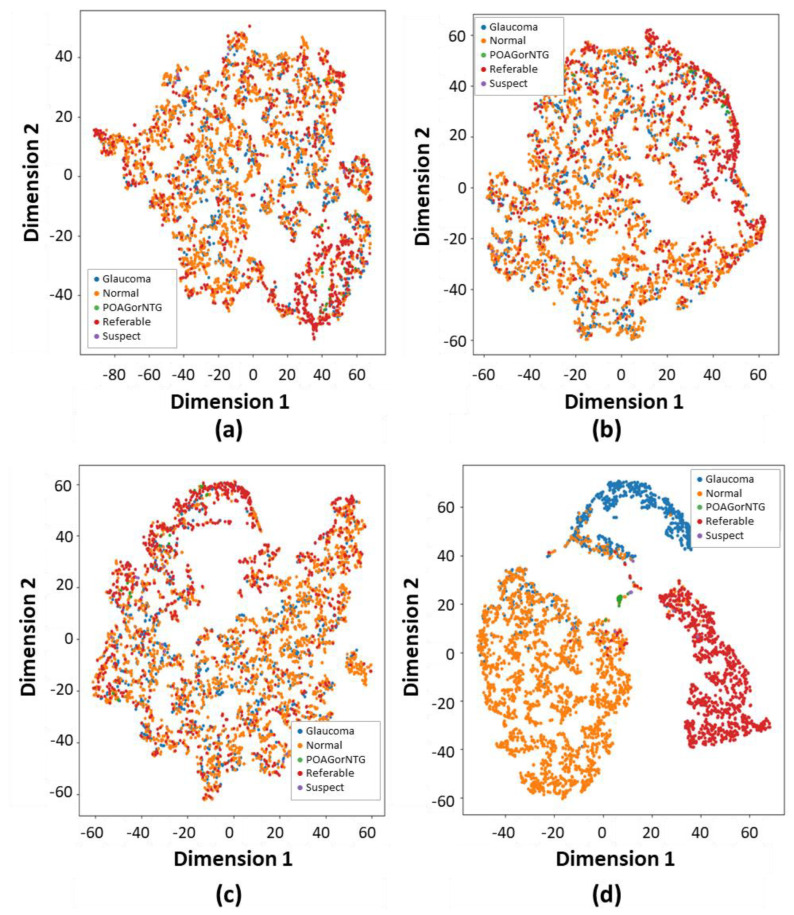
The verification of glaucoma classification is shown through the application of the t-distributed stochastic neighbor embedding (t-SNE) visualization technique. This method converts high-dimensional features into lower dimensions while retaining crucial features. The t-SNE technique enhances the accurate identification of each class through both depth-wise and color-wise strategies: (**a**) The Vit16 t-SNE method illustrates classification performance by distinguishing each class with a variety of colors. (**b**) The DeiT t-SNE-based approach categorizes each type of glaucoma. (**c**) The Swin transformer t-SNE method classifies each glaucoma category. (**d**) The performance of the proposed method’s t-SNE-based classification significantly exceeds that of other relevant studies.

**Figure 7:**
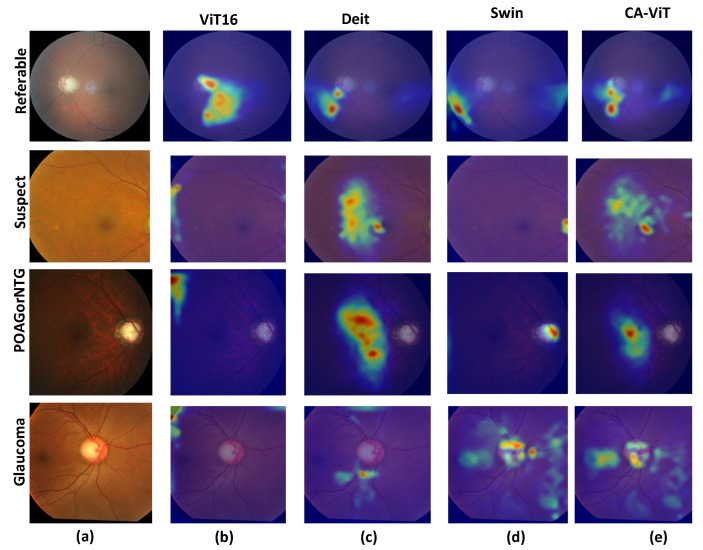
The attention heat maps for SOTA comparison: (**a**) The input disease types, which include four classes: POAG/NTG, glaucoma, referable, and suspect. (**b**) The attention heat maps for ViT16. (**c**) The attention heat maps for DeiT. (**d**) The attention heat maps for the Swin transformer. (**e**) The attention heat maps for CA-ViT (the proposed model).

Evaluation of the effectiveness and interpretability of the proposed method to enhance glaucoma classification was measured by qualitative analysis using multi-head attention heat maps and the t-SNE. These are presented in Figure 6 and Figure 7, respectively. We generated the attention heat maps using CA-ViT and visualized them using multi-head attention [21].

As demonstrated in Figure 6, we applied t-distributed stochastic neighbor embedding (t-SNE) to delve deeper into the model outputs. Specifically, t-SNE was applied to visualize the output feature spaces of the SOTA ViT models and the proposed model’s feature extractors for slice 32, as depicted in Figure 6, correspondingly. Upon comparing the spatial distributions of features between the normal, POAG/NTG, referable, suspect, and glaucoma classes, it was noted that the normal, glaucoma, and referable samples exhibited more condensed clustering within the feature space of contour-guided and augmented vision transformer compared to the SOTA models. This observation implies that the proposed contour-guided and augmented vision transformer demonstrates enhanced discriminative capabilities, proving to be more proficient in detecting and differentiating the intricate patterns specific to glaucoma in fundus images. The dataset we used exhibits a significant class imbalance, with only three dominant classes overshadowing others, such as suspect, mild, POAG/NTG, and various minor categories. This more accurately mirrors real-world clinical situations. These discrepancies highlight the enhanced generalizability and utility of the proposed model in precisely diagnosing glaucoma via multi-class fundus images.

Figure 7 shows the heat maps for each of the SOTA models. The generated heat maps for four disease types reveal more suspected disease areas. As shown in Figure 7b, the heat maps are located far from the optic disc and optic cup regions, indicating that the visualization results do not focus well on the required region. In Figure 7c, we obtain slightly better visualization results since the generated heat maps are very close to the optic disc and optic cup regions. Figure 7d shows slightly better results than the previous SOTA model, especially for the class POAG/NTG, which hits the focal points and achieves better results than CA-ViT. In Figure 7e, the proposed model shows better visualization results by focusing on the required areas for glaucoma classification. It clearly identifies suspected areas of glaucoma much better than the previous SOTA models across all classes. This is due to the multi-head attention and contour information available in the proposed model. The proposed CA-ViT model has a heightened level of interpretability due to the utilization of the multi-head attention mechanism inherent in vision transformers. This mechanism enables the model to focus on different areas of the input image, effectively capturing diverse features and details crucial for precise diagnosis [16]. It distinctly highlights areas requiring further scrutiny, as evidenced by the outcomes in the heat map.

Although the results achieved are encouraging because it is the first attempt to use contour and CVGAN for glaucoma classification, this study has some limitations. One of the main limitations is the absence of a multi-class glaucoma dataset, so all the results were evaluated only using SMDG, a combined dataset consisting of 19 publicly available datasets. Among these, the fundus images are not large enough, and some of the images are not labeled well. As a result, we achieved good classification results for three classes—normal, glaucoma, and referable—which were well represented in the dataset. However, the POAG/NTG and suspect classes need further representation and labeled data.

The CVGAN-based augmentation produces plausible fundus images when the given images are noise-free and of high quality. However, for low-contrast images and fuzzy borders, it fails to produce good-quality fundus images. To achieve good results with this approach, a large dataset is required. It would be better to use other mechanisms, such as diffusion models, which can produce good-quality images with fewer images under various conditions. In addition, the contour-guided information is also influenced by the quality of the input images. In the future, we will consider extracting high-quality contour information from noisy and low-quality images.

## 6. Conclusions

This paper presents a contour-guided and augmented vision transformer to enhance glaucoma classification in multi-class fundus images. Enhancing glaucoma classification is addressed by enlarging the data size using CVGAN-based augmentation techniques, as well as providing guidance information through a contour mechanism. The ViT backbone accepts both extracted contours and original images, and feature alignment is performed during training. The outcomes of the experimental analysis show good performance results with the efficacy of the framework, achieving a precision level of 93.0%, a recall rate of 93.08%, an F1 score of 92.9%, and an accuracy of 93%. These performance metrics exceed those attained by cutting-edge techniques, including the DeiT, Swin, and ViT16 models. Both the CVGAN-based augmentation and contour-guided modules significantly impact the model’s performance. In future investigations, we plan to extend our approach to OCT-based fundus or cross-sectional images for more efficient glaucoma detection, as well as apply it to other retinal diseases with similar pathology, such as Stargardt disease and Wet AMD. Additionally, we aim to incorporate diverse clinical data types, such as visual field tests and patient demographic information, to enhance diagnostic capabilities. Furthermore, investigating alternative data generation models, particularly for high-contrast fundus images, and integrating patch-to-patch attention mechanisms has the potential to enhance the efficacy of glaucoma diagnosis and classification, ultimately improving patient outcomes in glaucoma care.

## Figures and Tables

**Table 1:** Overview of utilized datasets sourced from the Standardized Multi-Channel Dataset for Glaucoma (SMDG-19), accessible on Kaggle [42].

Dataset	CRFO-v4	BEH	DRISHTI-GS	Eye-PACS	JSIEC	G1020	REFUGE	ODIA-ODIR	sjchoi86	ORIGA
Glaucoma	48	172	70	-	-	296	400	346	101	165
Normal	31	462	31	-	38	724	359	4302	300	485
POAG/NTG	-	-	-	-	-	-	40	-	-	-
Referable	-	-	-	3270	-	-	-	-	-	-
Suspect	-	-	-	-	11	-	-	14	-	-

**Table 2:** Data generation through augmentation and CVGAN in the SMDG dataset.

Disease	Original Train	Augmented	CVGAN	CVGAN & Augmented
Glaucoma	1598	4794	1000	5794
Normal	6732	8530	2000	10,530
POAG/NTG	40	120	20	140
Suspect	27	108	15	123
Referable	3270	6540	1000	7540

**Table 3:** Model architecture of CVGAN.

Component/Block	Layers/Operations	Details/Number of Blocks
Encoder	Convolutional Layer	Kernel: 1 × 1
	N + ReLU + Conv. 1 × 1	×1
	BN + ReLU + Conv. 3 × 3	×1
	BN + ReLU + Conv. 1 × 1	×1
	BN + ReLU + Conv. 1 × 1	×1
	Linear Layer	×1
Generator (G)	Linear Layer	×1
	N + ReLU + Conv. 1 × 1	×1
	BN + ReLU + Conv. 3 × 3	×1
	BN + ReLU + Conv. 1 × 1	×1
	BN + ReLU + Conv. 1 × 1	×1
	Linear Layer	×1
Discriminator (D)	Convolutional Layer	Kernel: 3 × 3, Stride: 1
	N + ReLU + Conv. 1 × 1	×1
	BN + ReLU + Conv. 3 × 3	×1
	BN + ReLU + Conv. 1 × 1	×1
	BN + ReLU + Conv. 1 × 1	×1
	Linear Layer	×1
	Softmax Layer	×1

**Table 4:** Model architecture of CA-ViT. MHA refers to multi-head attention.

Component/Block	Layers/Operations	Details/Number of Blocks
ViT backbone	6xTransformer Encoder	MHA, Linear, LayerNorm, Dropout
	6xTransformer Decoder	MHA, Linear, LayerNorm, Dropout
	Presence layer	Linear
	Query Embedding	Linear

**Table 5:** The parameters used for building both the proposed model and state-of-the-art (SOTA) models.

Model	Lr	Backbone	Weight Decay	Dropout
Ours	1.00 × 10^−5^	ResNet50	1 × 10^−6^	0.1
DeiT	1.00 × 10^−4^	-	1 × 10^−6^	0.1
Swin Transformer	1.00 × 10^−4^	-	1 × 10^−6^	0.1
ViT16	1.00 × 10^−4^	-	1 × 10^−6^	0.1

**Table 6:** Complexity comparison and performance results.

Method	Mean Accuracy (%)	Parameters (Millions)	FLOPs (GFLOPs)
ResNet50	72.61	23.5	4
ViT16	73.1	87.3	17.6
DeiT	75.03	85.6	17.5
Swin	76.0	86.7	15.4
CaiT	75.01	46.4	9.4
Ours	93.0	41.1	8

**Table 7:** Mean values are used to compare related works statistically.

Method	Precision	Recall	F1 Score	Accuracy
ResNet50	72.2	72.1	72.2	72.81
ViT16	73.4	73.4	73.2	73.31
DeiT	75.1	75.05	75.1	75.60
Swin Transformer	76.8	76.8	76.6	76.10
Ours	93.0	93.0	92.9	93.0

**Table 8:** The ablation results and *p*-values. The first column refers to different training configurations with the baseline ViT backbone: ViT + A (augmentation), ViT + G (generation), and ViT + C (contour).

Method	Precision	Recall	F1 Score	Accuracy	*p*-Value
ViT + A	79.4	79.4	79.2	79.4	-
ViT + G	83.1	83.7	83.2	83.4	<0.05
ViT + C	85.5	85.4	85.8	85.4	<0.05
ViT + A + G + C	93.0	93.0	92.9	93.0	<0.01

## Data Availability

In this study, we utilized SMDG, the Standardized Fundus Glaucoma Dataset, and publicly available datasets. These datasets can be made accessible upon request to the corresponding author, considering data privacy restrictions. The public datasets are accessible at the following link: https://www.kaggle.com/datasets/deathtrooper/multichannel-glaucoma-benchmark-dataset (accessed on 12 October 2023).

## Abbreviations

The following abbreviations are used in this manuscript:

SMDG	Standardized Fundus Glaucoma Dataset
DETR	Detection Transformer
ViT	Vision Transformer
CNN	Convolutional Neural Network
PAOG or NTG	Primary Open-Angle Glaucoma (PAOG) or Normal-Tension Glaucoma (NTG)
